# Investigating cutaneous tuberculosis and nontuberculous mycobacterial infections in a Department of Dermatology, Beijing, China: a comprehensive clinicopathological analysis

**DOI:** 10.3389/fcimb.2024.1451602

**Published:** 2024-08-23

**Authors:** Xin-Yu Wang, Qian-Nan Jia, Jun Li, He-Yi Zheng

**Affiliations:** Department of Dermatology, State Key Laboratory of Complex Severe and Rare Diseases, Peking Union Medical College Hospital, Chinese Academy of Medical Sciences and Peking Union Medical College, National Clinical Research Center for Dermatologic and Immunologic Diseases, Beijing, China

**Keywords:** skin diseases, infectious, cutaneous mycobacterial infections, *Mycobacterium tuberculosis*, nontuberculous mycobacterium, clinicopathologic study

## Abstract

**Background:**

Cutaneous tuberculosis (CTB) and nontuberculous mycobacteria (NTM) infections present considerable diagnostic and therapeutic challenges. This study aims to provide a comprehensive clinicopathological analysis of CTB and NTM infections.

**Methods:**

We conducted a retrospective analysis of 103 patients diagnosed with cutaneous tuberculosis (CTB) and nontuberculous mycobacteria (NTM) infections at a Beijing dermatology department from January 2000 to January 2024. Demographic, clinical, histological, and laboratory finding data were collected. Diagnostic methods and histopathological examination were recorded. Treatment regimens and outcomes were reviewed. Descriptive statistics were used to summarize demographic and clinical data, and continuous variables expressed as means and standard deviations (SD), and categorical variables as frequencies and percentages. Statistical analyses were conducted using SPSS version 25.0.

**Results:**

The cohort included 103 patients (40.8% males and 59.2% females), with a mean age of 51.86 years. Common clinical manifestations included nodules (97.1%), erythema (74.8%), and plaques (68.9%). Histological examination revealed hyperkeratosis (68.9%), parakeratosis (23.3%), and extensive neutrophil infiltration (95.1%) were observed. Acid fast bacteria (AFB) stains and nucleic acid tests exhibited respective positivity rates of 39.6% and 52.3%, respectively. Most patients were treated with a combination of three drugs; 77.1% of patients showed improvement, with the cure rate for CTB being 20.0%.

**Discussion:**

This study highlights the diverse clinical and histological presentations of CTB and NTM infections, emphasizing the need for comprehensive diagnostic approaches. The variability in treatment regimens reflects the complex management of these infections.

**Conclusion:**

The implementation of advanced molecular techniques and standardized treatment protocols is imperative for enhancing diagnostic precision and therapeutic outcomes.

## 1 Introduction

Infectious diseases in dermatology represent a diverse spectrum with myriad of clinical presentations and diagnostic challenges (Moffarah et al., 2016). Among these, skin infections caused by mycobacteria, particularly cutaneous tuberculosis (CTB) and nontuberculous mycobacterial (NTM) infections, stand as notable entities, posing complex diagnostic dilemmas and therapeutic complexities (Gonzalez-Santiago and Drage, 2015; Franco-Paredes et al., 2018; Sepulcri et al., 2023). CTB, a form of extrapulmonary tuberculosis, has seen a resurgence in recent years, presenting with varied clinical phenotypes, such as lupus vulgaris, scrofuloderma, and orificial tuberculosis. Similarly, NTM infections encompass a wide spectrum of species, and further compound the diagnostic challenge due to their diverse clinical manifestations and atypical presentations (Johansen et al., 2020).

While CTB and NTM infections have significant clinical impact, their management is often suboptimal (Gardini et al., 2022). This is largely due to a lack of awareness and the limited availability of specialized laboratory facilities and diagnostic tests. Reported studies on cutaneous mycobacterial infections are predominantly comprise scattered case reports that focus on either CTB or NTM, leading to a fragmented understanding of these often-overlooked infections. Many of these studies lack comprehensive analyses that integrate histopathological patterns, microbiological data, and treatment outcomes. The emergence of drug-resistant strains further complicates the management landscape, making it imperative to adopt a meticulous approach towards both diagnosis as well as treatment. This gap in comprehensive and integrated data underscores the necessity for a study that consolidates diverse aspects of CTB and NTM infections to provide a more complete and in-depth understanding (Baldwin et al., 2019).

To address these challenges, we have curated a repository of cases, and retrospectively analyzed patient demographics, clinical presentations, histopathological patterns, microbiological correlations, and treatment outcomes. The study endeavors to analyze a substantial pool of cases and improve understanding and awareness of CTB and NTM infections.

## 2 Methods

This retrospective, cross-sectional descriptive study was approved by the Ethical Committee of Peking Union Medical College Hospital (PUMCH) dated in April 2024. The study was conducted following the guidelines of the Health Insurance Portability and Accountability Act and all relevant laws (Shalowitz and Wendler, 2006).

Cases of CTB and NTM were identified from the electronic medical record system of our dermatology outpatient department, spanning from January 2000 to January 2024. A combination of clinical and microbiological assessments was performed to establish the diagnosis of CTB or NTM infection. All cases were confirmed through a series of standardized procedures by multiple senior dermatologists and pathologists. Any differing opinions were resolved through discussions with a third party. Any differences were resolved through joint discussions with a third researcher to avoid any potential bias. When available, patient data, including demographic information, acid-fast staining results, nucleic acid test results, pathological biopsy, and other clinical features were collected when available. For the outcome and prognosis, we defined completely or almost imperceptible changes, and mild pigmentation as cured outcomes, and moderate-severe pigmentation, localized scar, and remnant plaque as improved outcomes.

Categorical variables were presented in terms of percentages or proportions, while continuous variables were described using medians and quartile ranges. Statistical analysis was conducted using SPSS version 25.0 was conducted.

## 3 Results

### 3.1 Clinical and demographic characteristics of the patients

**Figure 1:**
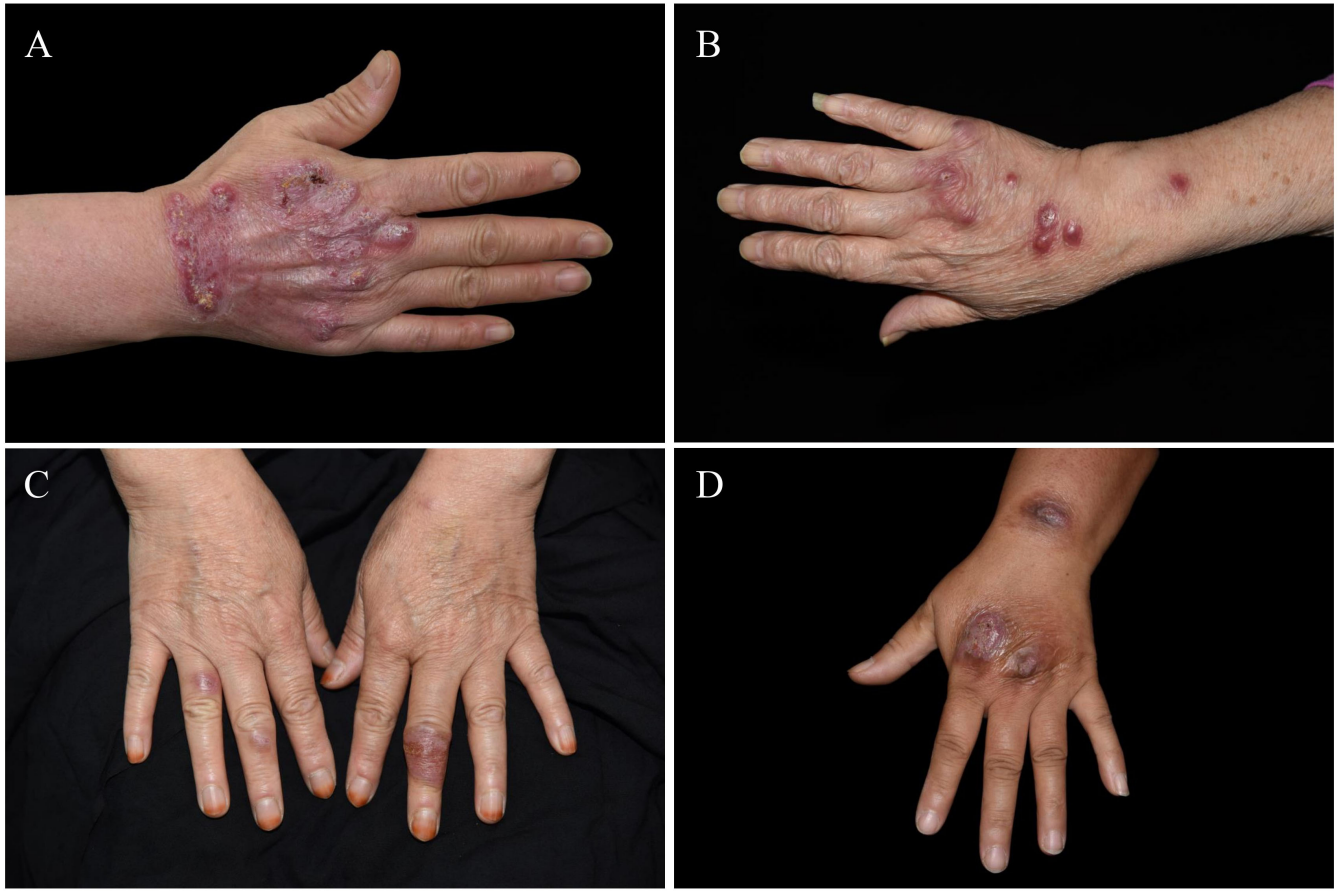
Clinical manifestations. **(A–D)** Chronic skin lesions primarily located in exposed areas.

The study population comprised 103 patients, with a higher proportion of females (59.2%, n=61) compared to males (59.2% vs. 40.8%). The mean age of the patients was 51.86 years (SD ± 16.25). Among the subgroups, the mean age of patients with CTB was 54.63 years (SD ± 15.65), those with rapid-growing mycobacteria (RGM, < 7 days for mature colony formation in solid media) had a mean age of 46.33 years (SD ± 16.02), and those with slow-growing mycobacteria (SGM, > 7 days for mature colony formation in solid media) had a mean age of 52.07 years (SD ± 15.54). According to the age distribution, the majority of patients (69.9%, n=75) were in the 30-60 age range. Patients in the age ranging from 60 years and older accounted for 23.3% of the study population, while those in 0-29 years age range constituted 6.8% (n=7). The most common clinical presentation was nodules, observed in 97.1% (n=100) of the patients. Erythema was present in 74.8% (n-77) of cases, and plaques were noted in 68.9% (n=71). Other clinical features included hyperplasia (37.9%, n=39), scales (23.3%, n=24), crusts (10.7%, n=11), erosions/ulcers (9.7%, n=10), abscesses (2.9%, n=3), and scars (14.6%, n=15). **Table 1** shows that the infection predominantly occurred in single sites, in 87.4% (n=90) of the patients, while involvement in multiple sites was seen in 12.6% (n=14). Most infections affected exposed body parts (92.2%, n=95), whereas in 7.8% (n=8) of the cases, non-exposed body parts were involved. Notably, all RGM infections affected exposed body parts. In the CTB group, nodules (80.0%, n=28), erythema (71.4%, n=25), and plaques (74.3%, n=26) were observed. Hyperplasia was observed in 37.1% (n=13) of CTB cases. For RGM, nodules were present in 69.2% (n=9), erythema in 53.8% (n=7), and plaques in 38.5% (n=5) of the cases. In the SGM group, nodules were the most prevalent at 88.0%, followed by erythema (81.8%, n=45) and plaques (72.7%, n=40). Hyperplasia was observed in 24.4% (n=22) of SGM cases. The skin lesions were dark red nodules and mostly located in the exposed areas. As shown in **Figures 1A–D**, most of the skin lesions are dark red nodules and are mostly located in the exposed areas.

**Table 1:** Demographic features of patients with cutaneous tuberculosis (CTB) and nontuberculous mycobacteria (NTM) infections.

Demographic features	CTB	NTM		Total
	N (%)	RGM	SGM	N (%)
		N (%)	N (%)	
Sex				
**Male**	15 (42.9)	4 (30.8)	23 (41.9)	42 (40.8)
**Female**	20 (57.1)	9 (69.2)	32 (58.1)	61 (59.2)
Age				
**Average (Mean ± SD)**	54.63 ± 15.65	46.33 ± 16.02	52.07 ± 15.54	51.86 ± 16.25
**0~29**	2 (5.8)	0 (0.0)	5 (9.1)	7 (6.8)
**30~60**	24 (68.6)	9 (69.2)	39 (70.9)	75 (69.9)
**≥60**	9 (25.6)	4 (30.8)	11 (20.0)	23 (23.3)
Clinical Presentations				
**Erythema**	25 (71.4)	7 (53.8)	45 (81.8)	77 (74.8)
**Plaque**	26 (74.3)	5 (38.5)	40 (72.7)	71 (68.9)
**Nodule**	28 (80.0)	9 (69.2)	44 (88.0)	100 (97.1)
**Abscessus**	2 (5.8)	0 (0.0)	1 (1.8)	3 (2.9)
**Erosion/Ulcer**	3 (8.6)	2 (15.4)	5 (5.6)	10 (9.7)
**Crust**	7 (20.0)	1 (7.7)	3 (9.1)	11 (10.7)
**Scale**	5 (14.3)	3 (23.1)	16 (29.1)	24 (23.3)
**Hyperplasia**	13 (37.1)	4 (30.8)	22 (24.4)	39 (37.9)
**Scar**	5 (14.3)	1 (7.7)	9 (16.4)	15 (14.6)
Site				
**Single site**	30 (85.7)	12 (92.3)	48 (87.3)	90 (87.4)
**Multiple sites**	5 (14.3)	1 (7.7)	7 (12.7)	14 (12.6)
**Exposed body parts**	32 (91.4)	13 (100.0)	47 (85.4)	95 (92.2)
**Non exposed body parts**	3 (8.6)	0 (0.0)	8 (14.6)	8 (7.8)

SGM, slowly growing mycobacteria; RGM, rapidly growing mycobacteria.

N, number.

SD, standard deviation.

### 3.2 Identification of mycobacterial isolates

Using different cultivation and sequencing methods, we identified 35 isolates of the *Mycobacterium tuberculosis complex*. Among the NTM, *M. marinum* was the most frequently isolated species in 37 cases. The SGM group included *M. kansasii* (five isolates), *M. avium* (three isolates), *M. intracellulare* (two isolates), and *M. haemophilum* (two isolates). Single isolates were identified for *M. szulgai*, *M. shigaense*, *M. parascrofulaceum*, *M. gordonae*, *M. colombiense*, *M. chimaera*, and *M. xenopi*. Notably, no isolates were found for *M. kumamotense*, *M. mageritense*, or *M. wolinskyi* in the SGM group. In the RGM category, *M. abscessus* was the most common species, with nine isolates. Other RGM species included *M. chelonae* (three isolates) and *M. fortuitum* (one isolate). There were no isolates for *M. immunogenum* or *M. neoaurum* in the RGM group (See more details in **Table 2**).

**Table 2:** Cutaneous tuberculosis (CTB) and nontuberculous mycobacteria (NTM) species isolated from the tissue of patients.

Mycobacterium species						
**Slowly growing mycobacteria**		***M. marinum***	***M. avium***	***M. intracellulare***	***M. haemophilum***	***M. szulgai***
	N	37	3	2	2	1
		***M. kumanotonense***	***M. kansasii***	***M. shigaense***	***M. parascrfulaceum***	***M. gordonae***
	N	0	5	1	1	1
		***M.colombiense***	***M. chimaera***	***M. mageritense***	***M. wolinskyi***	***M. xenopi***
	N	1	1	0	0	
**Rapidly growing mycobacteria**		***M. abscessus***	***M. chelonae***	***M. fortuitum***	***M. immunogenum***	***M. neoaurum***
	N	9	3	1	0	0
***M. tuberculosis* complex**	N	35				

N: Number of isolates.

### 3.3 Histopathological features of skin tissue biopsies

**Figure 2:**
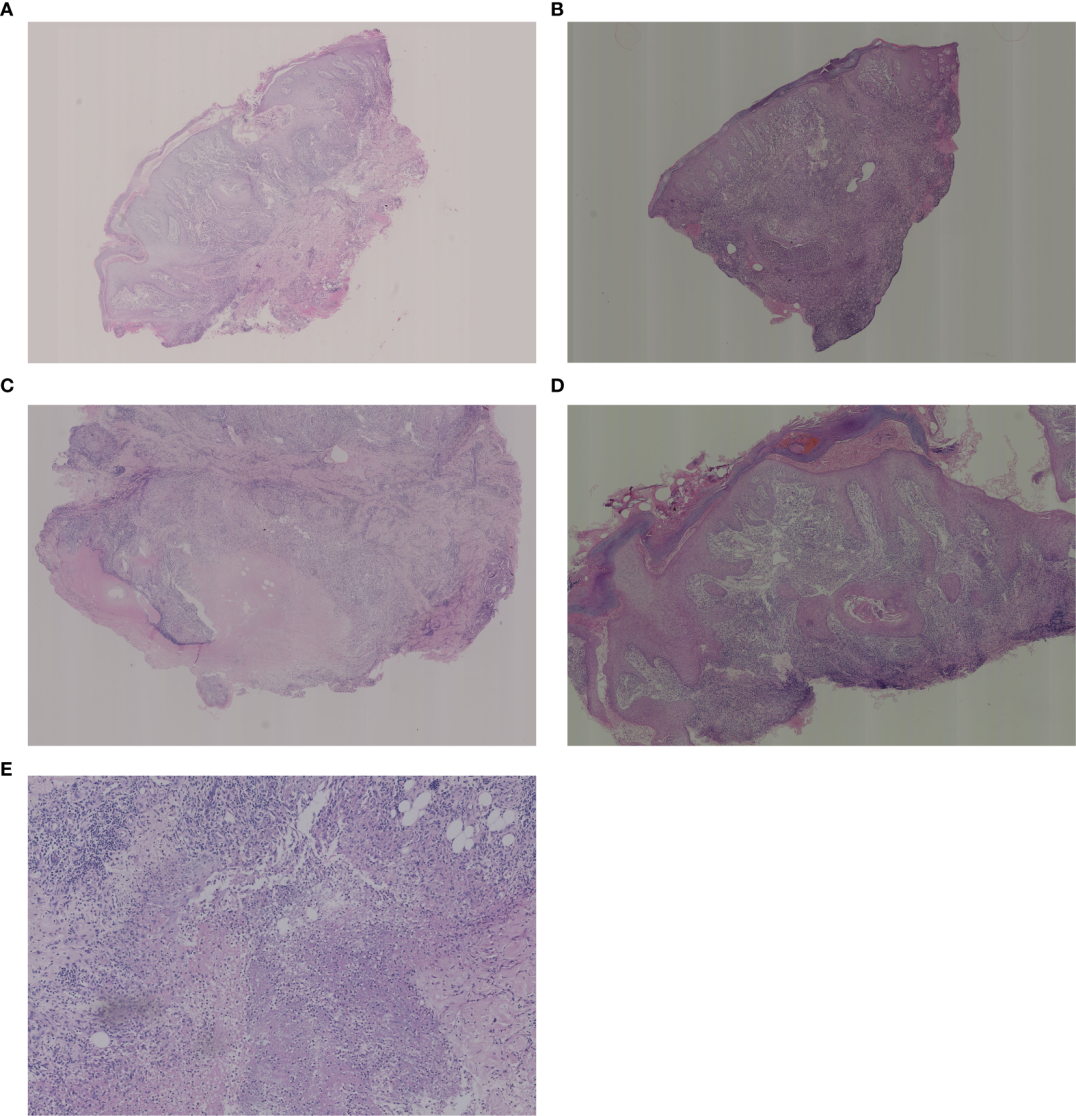
Histological examination of the biopsies. **(A)** Low magnification: pseudoepitheliomatous hyperplasia of the epidermis with areas of necrosis visible in the dermis, large numbers of neutrophils, surrounded by a dense infiltrate of lymphocytes, histiocytes, plasma cells and multinucleated giant cells. **(B)** Low magnification: superficial crusting, hyperkeratosis of the epidermis with hyperkeratosis, irregular thickening of the stratum spinosum, hyperextension of the rete ridges, and diffuse intradermal hyperplasia of small blood vessels with a large infiltrate of inflammatory cells, including lymphocytes, histiocytes, neutrophils, eosinophils, plasma cells, epithelioid cells, and multinucleated giant cells. **(C)** Medium magnification: crusting of the epidermis, localized ulcer formation, hyperkeratosis with hyperkeratosis, thickening of the epidermis with pseudoepitheliomatous hyperplasia. There are more nodules in the dermis with caseous necrosis in the center and infiltration of lymphocytes, epithelioid cells, histiocytes and plasma cells in the surrounding area. **(D)** Medium magnification: hyperkeratosis of the epidermis with hyperkeratosis, irregular thickening of the stratum spinosum, hyperextension of the rete ridges, massive vascular proliferation and dilatation of the dermis, and diffuse inflammatory cellular infiltrate including neutrophils, lymphocytes, histiocytes, plasma cells, epithelioid cells, and multinucleated giant cells are seen. **(E)** High magnification: large infiltration of lymphocytes, histiocytes, epithelioid cells and multinucleated giant cells in the deep and middle layers of the dermis. High magnification: tissue necrosis of the deep layer of dermis with numerous neutrophils and nuclear dust.

As shown in **Figure 2**, the results of some typical skin tissue pathological biopsies revealing various key features across different layers. Hyperkeratosis in the epidermis was observed in 71 cases (68.8%, n=71), parakeratosis in 24 cases (23.3%, n=24), and intercellular/inner cellular edema in 10 cases (9.7%, n=10). Acanthosis was noted in 39 cases (37.9%, n=39), elongated rete ridges in 9 cases (8.7%, n=9), dyskeratosis in 2 cases (2.0%, n=2), and liquefaction of the basal layer in 2 cases (2.0%, n=2), while 3 cases (2.9%, n=3) appeared nearly normal. In the dermis, neutrophil infiltration/nuclear dust was observed in 98 cases (95.1%, n=98), lymphocyte infiltration in 92 cases (89.3%, n=92), and plasma cell infiltration in 87 cases (78.6%, n=87). Histiocyte infiltration was seen in 59 cases (47.3%, n=59), multinucleated giant cells in 36 cases (35.0%, n=36), and small vessel proliferation/dilation in 32 cases (31.1%, n=32). Additionally, fibrous tissue degeneration/necrosis was observed in 17 cases (16.5%, n=17), noncaseating necrosis in 25 cases (24.3%, n=25), eosinophilic cell infiltration in 11 cases (10.7%, n=11), and caseous necrosis in 4 cases (3.9%, n=4). In the subcutis, nearly normal tissue was noted in 16 cases (15.5%, n=16), different inflammatory cell infiltration in 26 cases (25.2%, n=26), and this layer was not applicable in 61 cases (59.3%, n=61). Our analysis revealed diverse types of granulomatous inflammation and other significant histopathological features. Among the granulomatous inflammation cases, no granulomatous inflammation was seen in 36 cases, rheumatoid granulomatous inflammation in 3 cases, suppurative granulomatous inflammation in 11 cases, sarcoidal granulomatous inflammation in 1, and caseating granulomatous inflammation in 4 cases. Additionally, acute or chronic inflammation was noted in 64 cases and acanthosis or pseudoepitheliomatous hyperplasia was observed in 39 cases (See more details in **Table 3-1** and **Table 3-2**).

**Table 3-1:** Histological manifestations of patients with cutaneous tuberculosis (CTB) and nontuberculous mycobacteria (NTM) infections.

	Histological features	Cases with feature N (%)
**Epidermis**	**Approximately normal**	3 (2.9)
	**Hyperkeratosis**	71 (68.8)
	**Parakeratosis**	24 (23.3)
	**Dyskeratosis**	2 (2.0)
	**Intercellular/Inner cellular edema**	10 (9.7)
	**Acanthosis**	39 (37.9)
	**Liquefaction of basal layer**	2 (2.0)
	**Elongated rete ridges**	9 (8.7)
**Dermis**	**Multinucleated giant cell**	36 (35.0)
	**Histiocyte infiltration**	59 (47.3)
	**Lymphocyte infiltration**	92 (89.3)
	**Plasma cell infiltration**	87 (78.6)
	**Neutrophils infiltration/nuclear dust**	98 (95.1)
	**Eosinophilic cell infiltration**	11 (10.7)
	**Small vessel proliferation/dilation**	32 (31.1)
	**Fibrous tissue degeneration/necross**	17 (16.5)
	**Caseous necrosis**	4 (3.9)
	**Noncaseating necrosis**	25 (24.3)
**Subcutis**	**Approximately normal**	16 (15.5)
	**Different inflammatory cells infiltration**	26 (25.2)
	**Not applicable**	61 (59.3)

**Table 3-2:** Histological manifestations of patients with cutaneous tuberculosis (CTB) and nontuberculous mycobacteria (NTM) infections.

Characteristics	N
Granulomatous inflammation	
**Nil**	36
**Rheumatoid**	3
**Suppurative**	11
**Sarcoidal**	1
**Caseating**	4
**Acute or chronic inflammation**	64
**Acanthosis or pseudoepitheliomatous hyperplasia**	39

### 3.4 Summary of laboratory diagnostic methods

The summary of laboratory diagnostic methods for CTB and NTM infections are summarized in **Table 4**. The identification process included Ziehl-Neelsen staining for acid-fast bacilli confirmation, Polymerase Chain Reaction (PCR) and sequencing for species-specific identification, and Next-Generation Sequencing (NGS) for comprehensive genomic analysis, which facilitated precise species identification and detection of genetic markers associated with drug resistance. Acid-fast bacilli (AFB) staining was performed in 96 cases, 38 results were positive, with corresponding positivity rate of 39.6% (n=38). Nucleic acid tests were conducted on 65 cases, and 34 were positive, with 52.3% (n=34) positivity rate. Bacterial and fungal smears were examined in 36 cases, with 2 positives, yielding a 5.6% (n=2) positivity rate. Bacteria and fungi were cultured in 87 cases, with 29 positive results, giving a positivity rate of 33.3% (n=29). Anaerobic and aerobic bacteria were cultured for 26 cases, all of which were negative, resulting in a 0.0% positivity rate. In 86 cases, NGS was performed, with 26 positive results, yielding 30.2% (n=26) positivity rate. Periodic acid-Schiff (PAS) staining was performed on 26 cases, with 1 positive result (3.8%, n=1), suggesting a co-infection. Nocardia weak acid-fast stains and Nocardia cultures were performed in 28 cases, all yield yielding negative results. Actinobacteria were cultured in 24 cases, and all were negative.

**Table 4:** The summary of laboratory diagnosis methods of cutaneous tuberculosis (CTB) and nontuberculous mycobacteria (NTM) infections.

Methods	Total number (N)	Number of Positive (N)	Positivity rate
**Acid-fast bacilli (AFB) stains**	96	38	39.6%
**Nucleic acid tests**	65	34	52.3%
**Bacterial/Fungal smear**	36	2	5.6%
**Bacterial/Fungal culture**	87	29	33.3%
**Anaerobic/Aerobic bacterial culture**	26	0	0.0%
**Next generation sequence (NGS)**	86	26	30.2%
**Periodic Acid-Schiff (PAS) stain**	26	1^*^	3.8%
**Nocardia weak acid-fast stains**	28	0	0.0%
**Nocardia culture**	28	0	0.0%
**Actinobacteria culture**	24	0	0.0%

^*^: consider a co-infection.

N, number of cases.

### 3.5 Treatment choices and outcomes

**Table 5** presents the treatment choices and outcomes for the patients included in the study. For CTB, treatment regimens included the combination or alternation of two drugs in 14.3% of cases, three drugs in 71.4% (n=25), and four drugs in 14.3% (n=5) of cases, and an additional 5.7% (n=2) received other treatments. RGM patients were treated with two drugs in 7.7% (n=1) of cases, three drugs in 61.5% (n=8), and four drugs in 30.8% (n=4) of cases. SGM patients received one drug alone in 1.9% (n=1) of cases, two drugs in 14.5% (n=8), three drugs in 61.8% (n=34), and four drugs in 21.8% (n=12) of cases, with 5.5% (n=3) receiving other treatments. Treatment durations varied, with CTB patients receiving treatment for ≤3 months (11.4%, n=4), 3-6 months (57.1%, n=20), 6-12 months (28.6%, n=10), and >12 months (2.9%, n=1). Patients in the RGM group were treated for 3-6 months (23.1%, n=3) or 6-12 months (76.9%, n=9). SGM treatment durations were ≤3 months in 5.5% (n=3) of cases, 3-6 months in 34.5% (n=19), 6-12 months in 56.4% (n=31), and >12 months in 3.6% (n=2) of cases. Outcomes showed that 20.0% (n=7) of patients with CTB were cured, 77.1% (n=27) improved, and 2.9% (n=1) had poor outcomes, and there were no cases of mortality or lost follow-up. In the RGM group, 7.7% (n=1) were cured and 92.3% (n=12) improved. Outcomes in the SGM included 9.1% (n=5) cured, 83.6% (n=46) improved, 7.3% (n=2) with poor outcomes, and 3.6% (n=2) lost to follow-up.

**Table 5:** Treatment and outcome of cutaneous tuberculosis (CTB) and nontuberculous mycobacteria (NTM) infections.

Items	Number of cases		
	CTB N (%)	RGM N (%)	SGM N (%)
Treatments Choices			
**1 drug alone**	0 (0.0)	0 (0.0)	1 (1.9)
**2 drugs combination or alternation**	5 (14.3)	1 (7.7)	8 (14.5)
**3 drugs combination or alternation**	25 (71.4)	8 (61.5)	34 (61.8)
**4 drugs combination or alternation**	5 (14.3)	4 (30.8)	12 (21.8)
Others^*^	2 (5.7)	0 (0.0)	3 (5.5)
Treatment duration			
**≤3 months**	4 (11.4)	0 (0.0)	3 (5.5)
**3-6 months**	20 (57.1)	3 (23.1)	19 (34.5)
**6-12 months**	10 (28.6)	9 (76.9)	31 (56.4)
**>12months**	1 (2.9)	0 (0.0)	2 (3.6)
Outcomes			
**Cured^$^**	7 (20.0)	1 (7.7)	5 (9.1)
**Improved^&^**	27 (77.1)	12 (92.3)	46 (83.6)
**Poor**	1 (2.9)	0 (0.0)	2 (7.3)
**Mortality**	0 (0.0)	0 (0.0)	0 (0.0)
**Lost follow-up**	0 (0.0)	0 (0.0)	2 (3.6)

SGM, slowly growing mycobacteria; RGM, rapidly growing mycobacteria.

N, Number.

^*^: Oral medicines treatment coexists with other treatments, such as photodynamic therapy, hot compress, and surgical interventions.

^$^: Cured outcome means completely or almost imperceptible changes, mild pigmentation.

^&^: Improved outcome means moderate-severe pigmentation, localized scar, and remnant plaque.

## 4 Discussion

This study provides a comprehensive analysis of patients with CTB and NTM infections, focusing on the unique clinical and pathological features, diagnostic challenges, and treatment outcomes associated with these conditions. With this retrospective examination of a patient cohort from a leading department of dermatology in Beijing, China, we emphasize the variability in clinical presentations and the complexity of these infections. Our integration of traditional diagnostic methods highlights the importance of a multifaceted diagnostic approach. This study offers critical insights into the efficacy of current treatments, the need for individualized patient management, and provides valuable guidance for clinicians in the field.

The demographic analysis revealed that CTB and NTM infections were more prevalent in females compared to males, with an overall mean age of 51.86 years. This finding aligns with existing literature that suggests gender and age can influence susceptibility to mycobacterial infections (Henkle et al., 2017; Ricotta et al., 2021). Notably, most patients in this study were aged between 30 and 60 years, followed by those aged ≥60 years, and a smaller proportion in the 0-29 years range. These findings are consistent with earlier published studies indicating a higher likelihood of middle-aged and elderly populations presenting with cutaneous mycobacterial infections, potentially attributed to cumulative environmental exposure and declining immune function with age (Philips et al., 2019; Fujishima et al., 2022). The most common manifestation included the nodules, underscoring the importance of considering mycobacterial infections in the differential diagnosis of nodular skin lesions. Other notable features included erythema, plaques, hyperplasia, and scales. These diverse presentations highlight the variable clinical spectrum of, necessitating a high index of suspicion and thorough diagnostic workup. Our findings underscore the importance of focusing on chronic skin lesions in exposed areas, especially in patients with a history of trauma, surgery, or cosmetic procedures, as these factors contribute to increased risk of cutaneous infections (Shen et al., 2024; Tabaja et al., 2024). In these cases, mycobacterial infections should be a key differential diagnosis to ensure prompt and appropriate treatment. The presence of a variety of NTM species in cutaneous infections highlights the clinical importance of accurate diagnosis and species identification, as implications of different mycobacteria may varying in terms of treatment and prognosis. *M. marinum*, the most frequently isolated species in our study, is commonly associated with skin and soft tissue infections, particularly in individuals exposed to aquatic environments (Tsiolakkis et al., 2023). Susceptibilities of different species of NTM to antibiotics exhibit variations, necessitating species-specific treatment regimens. For example, *M. marinum* infections often require a combination of antibiotics, such as rifampin and ethambutol, sometimes along with clarithromycin. However, other NTM species, such as *M. abscessus*, pose significant treatment challenges due to their intrinsic resistance to many common antibiotics (Johansen et al., 2020). These infections typically involve a prolonged course of multiple antibiotics, such as macrolides and aminoglycosides, and may sometimes require surgical intervention for the removal of infected tissue.

The histopathological features observed in CTB and NTM infections offer crucial insights into the underlying inflammatory processes and immune responses. Each feature is a marker for specific biological activities occurring within the infected tissue and provides a comprehensive understanding of the host-pathogen interaction. The first responders to infection include neutrophils, their high prevalence in the tissue samples indicates an acute inflammatory response, suggesting active infection and ongoing immune defense mechanisms attempting to control the mycobacterial pathogens. Lymphocytes are commonly seen in histopathological examinations and play a crucial role in adaptive immunity. Their presence indicates a chronic immune response, where the body recognizes mycobacterial antigens and incites a targeted defense. The chronic nature of mycobacterial infections often requires sustained lymphocyte activity to manage and contain the infection. Multinucleated giant cells are a hallmark of granulomatous inflammation. These cells formed by macrophage fusion in response to persistent infection, attempt to engulf and digest the mycobacteria Nonetheless, mycobacteria have evolved mechanisms to survive within these cells. The presence of giant cells indicates the attempt of the body to isolate and contain the infection within granulomas. Histiocytes, or tissue macrophages, are central to granuloma formation. They ingest and present mycobacterial antigens to lymphocytes, initiating and sustaining the immune response. Histiocytes also release cytokines that recruit other immune cells to the infection site and facilitate the formation of granulomas. Histiocyte infiltration signifies an ongoing immune response to manage and limit the spread of mycobacteria (Terziroli et al., 2018). These findings corroborate the histological diversity observed in previous studies and emphasize the importance of biopsy and histopathological examination in the diagnosis of CTB and NTM infections (Asai, 2017). The histopathological features identified provide valuable information for diagnosing and treating mycobacterial infections. Recognizing the patterns of neutrophil and lymphocyte infiltration, and granuloma formation, can help differentiate CTB and NTM infections from other skin conditions. This differentiation of CTB and NTM infections is critical as it guides the choice of treatment regimens. Histological investigation of the interactions between mycobacteria and host immune cells can unravel mechanisms of immune evasion and persistence of mycobacteria within granulomas. Furthermore, understanding the signaling pathways involved in the formation and maintenance of granuloma can aid in the development of targeted therapies to modulate the immune response and enhance bacterial clearance.

Confirmation of mycobacterial infections and differentiating them from other conditions with similar presentations through laboratory diagnosis is crucial. The study demonstrates varying positivity rates across different diagnostic methods. Nucleic acid tests, with a positivity rate of 52.3%, offer the highest diagnostic yield and are increasingly preferred for their sensitivity and specificity (Luetkemeyer et al., 2014). Bacterial and fungal cultures had a positivity rate of 33.3%, but the time required for growth can delay diagnosis and treatment. NGS had a positivity rate of 30.2%, highlighting its potential for more accurate and rapid identification of mycobacterial species. Nowadays, the integration of cell-free DNA (cfDNA) with NGS technology allows for the rapid and efficient detection of Mycobacterium tuberculosis-specific gene fragments from skin samples, facilitating more sensitive and specific early diagnosis (Shao et al., 2020). NGS can comprehensively analyze the genome of M. tuberculosis present in cfDNA samples, identifying gene mutations associated with drug resistance. This capability for personalized treatment, is of paramount importance as it aids in selecting the most effective anti-tuberculosis drugs based on the specific drug susceptibility profile of the patient, thereby enhancing treatment efficacy (Blauwkamp et al., 2019). By regularly monitoring the levels of M. tuberculosis cfDNA in samples, the disease progression and the treatment effectiveness can be dynamically assessed. A decline in cfDNA levels during treatment indicates therapeutic success, whereas stable or rising levels may suggest treatment failure or the development of drug resistance. This approach enables real-time evaluation and adjustment of treatment regimens (Hogan et al., 2021). Therefore, incorporating advanced diagnostic techniques into routine practice is essential for optimizing patient care and improving the management. However, the diagnosis of CTB and NTM remains challenging, primarily due to the absence of diagnostic methodologies that offer both high sensitivity and specificity (Opota et al., 2021). Presently, no single diagnostic test can reliably and conclusively identify these infections, necessitating a more integrative approach to diagnosis. For an accurate diagnosis, it is imperative to undertake a comprehensive evaluation that encompasses several critical aspects. These include a detailed review of the patient’s medical history, comprehensive assessment of clinical manifestations, meticulous pathological examination, and precise interpretation of laboratory findings. Additionally, empirical treatment responses can provide invaluable diagnostic insights, further informing the clinical decision-making process.

Treatment regimens varied significantly, reflecting the complexity of managing CTB and NTM infections. As we summarized in our previous study, these variabilities in treatment duration and regimen highlight the need for individualized therapy based on the patient’s response and the specific species involved (Wang et al., 2023). Hence, the treatment choice should depend on the species, susceptibility pattern, severity, and extent of infection. Alternative treatment options in skin conditions include surgical interventions, phototherapy, and heat application. A safer, more effective, and broader-spectrum treatment strategy for CTB and NTM infections is needed. Most existing treatment strategies are based on case reports, small case series, and expert opinions. There is a pressing need for high-quality randomized controlled trials (RCTs) to evaluate the efficacy and safety of different modalities for the treatment of CTB and NTM infections (Wang et al., 2023; Cimino et al., 2024). Recent studies have explored new antibiotics and novel combinations of existing drugs to enhance efficacy and reduce resistance. For instance, host-directed therapies (HDTs), aiming to enhance the immune response of the host against mycobacterial infections. Immunomodulatory agents like interferon-γ and tumor necrosis factor α inhibitors are under investigation for their ability to modulate the immune response and improve treatment outcomes (Chung et al., 2018; Crilly et al., 2020; Da et al., 2020). Future research should focus on well-designed RCTs to establish evidence-based guidelines, explore novel therapeutic agents, and develop preventive strategies such as vaccines. In our study, poor outcomes in a small percentage of cases, alongside the incidence of lost follow-up, highlights the challenges in achieving complete resolution and maintaining long-term follow-up. Successful management of CTB and NTM infections often requires prolonged treatment with multiple antimicrobial drugs, necessitating vigilant monitoring for adverse effects and resistance development. Regular follow-up is essential to ensure adherence to the treatment regimen, monitor response, and adjust therapy as needed.

At the forefront of current research efforts are emerging treatments for CTB and NTM infections, including new antimycobacterial agents and adjunctive therapies. The development and clinical testing of these innovative modalities aim to enhance the efficacy of existing treatment regimens, reduce treatment duration, and minimize adverse effects (Casanova et al., 2024; Dartois and Dick, 2024). Successful outcomes from these trials might lead to the establishment of novel standard-of-care protocols, offering more effective and patient-friendly treatment options. Other important areas of research include the understanding of host-pathogen interactions and the role of the immune system in controlling mycobacterial infections (Poerio et al., 2022). Research on immune responses and potential biomarkers for disease activity and treatment response can lead to the development of personalized treatment strategies and improve prognostic assessments (Bittencourt et al., 2021). Efforts are also needed to develop vaccines to prevent CTB and NTM infections or reduce disease severity. These vaccines aim to prime the immune system to recognize and respond more effectively to mycobacterial pathogens, reducing the incidence and impact of infections (Shah et al., 2019).

## 5 Limitations

While this study provides valuable insights into the characteristics of CTB and NTM infections, it has several limitations. First, the retrospective design, relying on the accuracy and completeness of medical records, introduces potential biases such as selection and information bias. Additionally, the variability in treatment regimens among patients complicates the ability to draw definitive conclusions about the efficacy of specific protocols. Inconsistencies in follow-up duration may also affect reported outcomes, necessitating longer and more standardized follow-up periods for accurate assessment. The lack of detailed data on drug resistance and relapse is a significant limitation, as these factors are critical for understanding long-term treatment outcomes and managing these infections effectively. Histopathological interpretation potentially varied between pathologists, highlighting the need for standardized criteria and interobserver reliability assessments. Finally, the research focused primarily on clinical and histopathological features, which could provide further insights into disease mechanisms and inform personalized treatment strategies.

## 6 Conclusion

This study provides a detailed overview of the characteristics of patients with CTB and NTM infections. The findings highlight the importance of comprehensive diagnostic approaches in accurately identifying and managing these infections. The variability in treatment regimens and outcomes highlights the need for individualized treatment strategies and ongoing research into new diagnostic and treatment approaches. By integrating recent advances and exploring innovative approaches, the management and prognosis of patients with CTB and NTM infections can be significantly improved.

## Data Availability

The original contributions presented in the study are included in the article/supplementary material. Further inquiries can be directed to the corresponding authors.
