# Corrigendum: Investigating cutaneous tuberculosis and nontuberculous mycobacterial infections in a Department of Dermatology, Beijing, China: a comprehensive clinicopathological analysis

**DOI:** 10.3389/fcimb.2024.1532147

**Published:** 2024-12-05

**Authors:** Xin-Yu Wang, Qian-Nan Jia, Jun Li, He-Yi Zheng

**Affiliations:** Department of Dermatology, State Key Laboratory of Complex Severe and Rare Diseases, Peking Union Medical College Hospital, Chinese Academy of Medical Sciences and Peking Union Medical College, National Clinical Research Center for Dermatologic and Immunologic Diseases, Beijing, China

**Keywords:** skin diseases, infectious, cutaneous mycobacterial infections, *Mycobacterium tuberculosis*, nontuberculous mycobacterium, clinicopathologic study

In the published article, there was an error in the article title. Instead of “Investigating cutaneous tuberculosis and nontuberculous mycobacterial infections a Department of Dermatology, Beijing, China: a comprehensive clinicopathological analysis”, it should be “Investigating cutaneous tuberculosis and nontuberculous mycobacterial infections in a Department of Dermatology, Beijing, China: a comprehensive clinicopathological analysis”.

The authors apologize for this error and state that this does not change the scientific conclusions of the article in any way. The original article has been updated.

